# The Multifaceted Roles of MSCs in the Tumor Microenvironment: Interactions With Immune Cells and Exploitation for Therapy

**DOI:** 10.3389/fcell.2020.00447

**Published:** 2020-06-19

**Authors:** Andrea Papait, Francesca Romana Stefani, Anna Cargnoni, Marta Magatti, Ornella Parolini, Antonietta Rosa Silini

**Affiliations:** ^1^Centro di Ricerca E. Menni, Fondazione Poliambulanza Istituto Ospedaliero, Brescia, Italy; ^2^Department of Life Science and Public Health, Università Cattolica del Sacro Cuore, Rome, Italy; ^3^Fondazione Policlinico Universitario “Agostino Gemelli” IRCCS, Largo A. Gemelli, Rome, Italy

**Keywords:** perinatal, placenta, mesenchymal stromal cells, cancer, tumor microenvironment, inflammation, immunosurveillance, immunoediting

## Abstract

The tumor microenvironment (TME) plays a critical role in tumorigenesis and is composed of different cellular components, including immune cells and mesenchymal stromal cells (MSCs). In this review, we will discuss MSCs in the TME setting and more specifically their interactions with immune cells and how they can both inhibit (immunosurveillance) and favor (immunoediting) tumor growth. We will also discuss how MSCs are used as a therapeutic strategy in cancer. Due to their unique immunomodulatory properties, MSCs isolated from perinatal tissues are intensely explored as therapeutic interventions in various inflammatory-based disorders with promising results. However, their therapeutic applications in cancer remain for the most part controversial and, importantly, the interactions between administered perinatal MSC and immune cells in the TME remain to be clearly defined.

## Introduction

It is now clear that the tumor microenvironment (TME) is essential in tumorigenesis (Mantovani et al., 2008). Different immune players are found in the TME, such as those pertaining to innate immunity [i.e., macrophages, neutrophils, mast cells, myeloid-derived suppressor cells (MSDCs), dendritic cells (DCs), and natural killer (NK) cells] and those of adaptive immunity (T and B lymphocytes) (Turley et al., 2015). Immune cells within the TME give rise to an inflammatory response, which plays a fundamental role in all stages of tumor growth (Mantovani et al., 2008), where on one hand it can stimulate an anti-tumor immune reaction (immunosurveillance), and on the other hand it can be exploited to promote cancer progression (immunoediting) (O’Donnell et al., 2019). In addition, the TME is also composed of tumor stroma cells that have multifaceted interactions with immune cells [mesenchymal stromal cells (MSCs), fibroblasts, endothelial cells, and pericytes] (de Visser et al., 2006). The heterotypic interactions that occur between these different players are highly complex and involve the exchange of molecules such as cytokines, chemokines, and mitogens, which are crucial in determining a pro- or anti-tumor outcome.

MSCs isolated from bone marrow, adipose tissue, and fetal tissues are also intensely explored as therapeutic interventions in various inflammatory-based disorders, including cancer. The rationale behind their use lies within their remarkable migration ability, which can be employed for therapeutic strategies, such as the delivery and local secretion of bioactive factors and/or for the tumor-specific delivery of chemotherapeutic agents. The challenges that lie ahead of the transition from bench to bedside involve a superior understanding of the interplay between the tumor, its microenvironment, and administered/exogenous MSC. Indeed, once caught in the tumor net, MSCs can be hijacked by the malignant cells and manipulated for the tumor’s own advantage. Nonetheless, many studies have also ascertained that exogenous MSCs restrain tumor progression.

In this review, we will discuss MSCs in the context of the TME and more specifically immune cells, and the interactions between MSCs and immune cells within the TME that can either inhibit or favor tumor growth. We also discuss how MSCs can be used as a therapeutic strategy in cancer, with a particular focus on perinatal MSCs, the latter of which has been our research interest for over 15 years.

## The Immune System in Cancer

### Tumor Suppression: Immunosurveillance

The importance of immune cells (and inflammation) in controlling and contributing to tumor progression has been acknowledged for decades. Both innate and adaptive immune components actively patrol the body to identify and eradicate incipient tumor cells, and the description of tumor-infiltrating lymphocytes with effector and memory functions within primary tumors and their metastases have been largely described (Motz and Coukos, 2013). The presence of an immune infiltrate is generally associated to good prognosis; however, this is largely dependent on the tumor type, location of the cells, and their state of activation (Barnes and Amir, 2018). Tumor cells express mutated neo-antigens, non-mutated antigens encoded by genes overexpressed by cancer cells, or antigens encoded by differentiation genes related to the cancer’s tissue of origin. DCs process tumor antigens and promote their presentation to T cells, generating an anti-tumor response. Within T cells, CD8^+^ cytotoxic T cells remain the most potent mediators of anti-tumor immunity, and a response directed by either CD4^+^ T helper 1 (Th1) cells or Th17 cells promote CD8^+^ effector T cell responses (Martin-Orozco et al., 2009). Their efficacy is suggested by the fact that a large infiltration of CD8^+^ cytotoxic T cells is linked to a favorable prognosis in different types of tumors, such as breast, ovarian, lung, and colorectal cancer (Barnes and Amir, 2018).

Several studies have underlined the critical and distinct roles for γδ T cells, and αβ T cells (Girardi et al., 2003), as well as NK and NKT cells, in immunosurveillance against cancer (Smyth et al., 2000; Dunn et al., 2004) in which interferon-γ (IFN-γ), perforin, and IFN-α/β represent key factors involved in triggering cancer cell death (Smyth et al., 2000; Dunn et al., 2004). B lymphocytes can also promote anti-tumor immunity, as shown by the detection of antibodies against tumor antigens in the serum of cancer patients (Yuen et al., 2016). Antibodies have multifaceted functions in the TME. They can activate the complement cascade, alter the function of their targets on cancer cells, opsonize tumor cells for antigen presentation by DC, or contribute to NK cell-mediated tumor killing via antibody-dependent cell-mediated cytotoxicity (Yuen et al., 2016). Classically, T-cell priming occurs in tumor-draining secondary lymph nodes; however, spontaneously organized tertiary lymphoid organ structures can be encountered in tumors, suggesting a local anti-tumor defense, with a T-cell education that can occur within the tumor stroma, thus suggesting a local T-cell education and anti-tumor defense. Again, their presence is associated with favorable prognosis in most solid malignancies (Allen et al., 2017; Sautes-Fridman et al., 2019).

### Tumor Promotion: Immunoediting

The surveillance of immune cells seems to be achieved only at the earliest stages of tumorigenesis. In the majority of established tumors, the presence of lymphocytes is not sufficient for curbing tumor growth. In addition, whereas controlled inflammation may be involved in the eradication of tumors, excessive inflammation may facilitate the transformation process. These considerations have given rise to the concept referred to as immunoediting (Dunn et al., 2004) whereby tumor cells constantly edit and modulate the host antitumor immune response, thus shaping tumor immunogenicity and clonal selection and ultimately shifting the balance in favor of tumor growth. Furthermore, in the initial phases of tumor expansion and progression, tumor cells contribute to creating an inflammatory milieu (Coussens and Werb, 2002) by producing and releasing high amount of molecules that either directly or indirectly induce cell proliferation, recruit inflammatory cells that further support tumor growth (Ren et al., 2012; Greten and Grivennikov, 2019), and increase production of reactive oxygen species (Coussens and Werb, 2002; Landskron et al., 2014).

**FIGURE 1:**
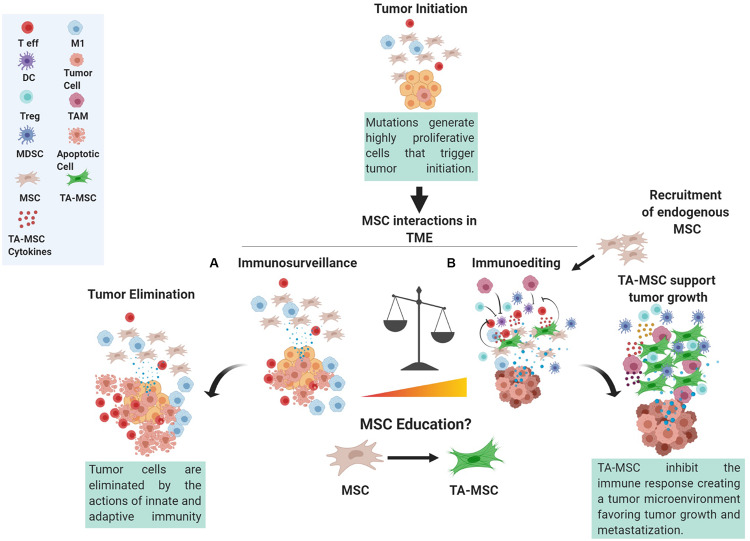
The role of MSC in the tumor microenvironment. Mutations occurring in cells can trigger tumor initiation. During the initial steps, tumor cells proliferate, produce, and release antigens that can be recognized by the immune system. At this point, a subtle balance between the action of immunosurveillance performed by both innate and adaptive immunity counteracts the actions of tumor cells giving rise to a complex network of interactions within the surrounding tumor microenvironment (TME). Indeed, MSCs in contact with the tumor cells, referred to as tumor-associated MSC (TA-MSC), could be considered a crucial axis of these interactions by altering tumor cell phenotype and the release of cytokines and other molecules that can in turn trigger the education of immune cells and of other progenitor cells present in the TME. These interactions give rise to MSC involvement in immunosurveillance **(A)** or immune-editing **(B)**, whereby MSCs can stimulate an anti-tumor immune reaction or skew immune cells toward an immune suppressive phenotype thus favoring immune evasion. T eff, T effector; DC, dendritic cells; Treg, T regulatory cell; MDSC, myeloid derived suppressor cell; MSC, mesenchymal stromal cell; TA-MSC, tumor associated MSC; M1, macrophage type M1; TAM, tumor associated macrophage.

However, within the TME, there are different cells, such as MSCs, that can impact the immune response (Figure 1), the latter of which has a profound impact on tumor progression.

## MSCs in the TME

Regardless of the biological setting, MSCs perform supportive and regenerative functions, thus nourishing the surrounding environment, and MSCs located in the same tissue but exposed to different stimuli may adjust their behavior accordingly, as demonstrated by three-dimensional models that study the interactions between MSCs, tumor cells, and immune cells (Poggi et al., 2018). Indeed, stromal cell heterogeneity has been attributed to stage of development, tissue of origin, and tissue microenvironment (Lynch and Watt, 2018).

Generally, MSCs constitute an important component of the tumor stroma (herein referred to as tumor-associated MSC, TA-MSC). Faithful to their intrinsic role as supportive cells (Caplan and Dennis, 2006), MSCs have been shown to act in a multi-modal fashion and to directly or indirectly contribute to tumor survival and progression, although evolving data also argue for a role in tumor regression (Oloyo et al., 2018).

Before digging into MSC functions within the TME, it is important to clearly define MSCs, their origin, and what attracts them to malignancy; in the following sections, we will discuss these aspects.

### MSC Phenotype and Origin

MSCs are primordial multi-potent fibroblast-like cells first described in the bone marrow by Friedenstein et al. (1968, 1974), with the capacity to regenerate bone marrow stroma at heterotypic sites (Sacchetti et al., 2007). These cells are now believed to reside in almost all fetal and post-natal organs (da Silva Meirelles et al., 2006; Crisan et al., 2008; Marrazzo et al., 2016), bearing tissue-specific transcriptional profiles and lineage capabilities (Sacchetti et al., 2016).

Owing to the lack of unequivocal markers of endogenous MSC, our knowledge of the properties of these cells has its roots in *in vitro* studies. Indeed, the difficulties encountered in discriminating naïve MSCs from other cell populations *in vivo*, such as fibroblasts (Soundararajan and Kannan, 2018) and pericytes (Cano et al., 2017), persist also in cancer. As a matter of fact, cancer-associated fibroblasts (CAFs) coexist as heterogeneous population comprising cells with overlapping phenotypes, whose proportion may vary according to the tumor, and of which MSCs constitute only a fraction, thus often generating inconsistency and confusion within the field.

As bone marrow-derived cells constitute the earliest and the best characterized MSC (BM-MSC), most of the studies exploited their phenotypic pattern to isolate mesenchymal stromal-like cells from various sources, including cancer. In 2006, a position paper by the International Society for Cellular Therapy (ISCT) (Dominici et al., 2006) delineated a set of minimal criteria to identify human BM-MSC *in vitro*, such as (a) plastic adherence; (b) positive expression of CD105, CD73, and CD90, and absence of the hematopoietic antigens CD45, CD34, CD14 or CD11b, CD79α or CD19, and HLA-DR; and (c) multi-lineage differentiation. The BM-MSC phenotype has often been used as a reference for the TA-MSC phenotype.

MSCs have been isolated from different types of human malignancies, including head and neck (Kansy et al., 2014), glioma (Behnan et al., 2014; Hossain et al., 2015; Shahar et al., 2017; Svensson et al., 2017), breast metastasis (Gonzalez et al., 2017), cervical (Avila-Ibarra et al., 2019), ovarian (McLean et al., 2011; Coffman et al., 2019; Naour et al., 2019), lung (Galland et al., 2017), prostate (Hughes et al., 2019), neuroblastoma (Pelizzo et al., 2018), and colorectal (Zhang et al., 2018) cancer. Even if the MSCs in these studies adhered to the ISCT phenotype (Dominici et al., 2006), it is important to bear in mind that tumor cells can also exhibit a mesenchymal-like phenotype, making it difficult, or even impossible, to discriminate them from MSCs.

MSCs have often divided the scientific community concerning many aspects of their biology. In cancer, one of the questions that is still a matter of debate is whether TA-MSCs originate from the local microenvironment or are recruited from remote locations of the body. Several studies have indeed shown that CAFs may arise from local fibroblasts and MSCs (Udagawa et al., 2006; Erez et al., 2010; Kojima et al., 2010; Sharon et al., 2015; Arina et al., 2016), from adipocytes and adipocyte-associated stromal cells (Zhang et al., 2009, 2012; Dirat et al., 2011; Bochet et al., 2013), from the recruitment of BM-MSCs into the tumor tissue (Direkze et al., 2003, 2004; Mishra et al., 2008; Spaeth et al., 2009; Quante et al., 2011; Kidd et al., 2012; Kalluri, 2016; Borriello et al., 2017; Raz et al., 2018; Bhagat et al., 2019), from endothelial-to-mesenchymal transition (EndMT) of tumor-associated endothelial cells (Zeisberg et al., 2007), and from epithelial-to-mesenchymal transition (EMT) of non-malignant or malignant epithelial cells (Radisky et al., 2007). Several pathways have been reported to drive the transition of MSCs into CAFs, such as epigenomic reprogramming via lactate in pancreatic cancer (Bhagat et al., 2019), exposure to oxidative stress in breast cancer (Toullec et al., 2010), C-X-C chemokine receptor type 6 (CXCR6) (Jung et al., 2013), and transforming growth factor (TGF)β1 (Barcellos-de-Souza et al., 2016) signaling in prostate cancer, and a supportive role for CD44 in preserving a functional phenotype of CAF has been described (Spaeth et al., 2013). Once within the tumor, BM-MSCs become activated fibroblasts, express markers typical of myofibroblasts such as alpha smooth muscle actin (α-SMA), vimentin, or fibroblast specific protein (FSP) (Quante et al., 2011) and contribute to extracellular matrix remodeling (Dumont et al., 2013), eventually fine-tuning their activation state according to the clues they perceive (Augsten, 2014). Activated fibroblasts further display enhanced proliferative and migratory capacity as a consequence of TGFβ1 (Lohr et al., 2001) and platelet-derived growth factor (PDGF) (Vignaud et al., 1994; Cheng et al., 2013) stimulation, among others. To sustain this new highly energetic and extremely expensive “lifestyle,” CAF undergo a metabolic reprogramming, thus relying upon aerobic glycolysis, often in a cooperative interaction with tumor cells (Fiaschi et al., 2012; Lyssiotis and Kimmelman, 2017).

Endowed with the capacity to promote or restrain tumor growth, stromal cells exert their action by manipulating several components of the TME, and in particular immune cells.

### MSC Crosstalk With Immune Components of the Inflammatory Niche in Solid Cancer

Several studies have underlined the relevance of MSC in supporting tumorigenesis targeting several components and pathways of the TME. Much evidence supports MSCs action on tumor immune cells through their paracrine actions and the ability to modify the microenvironment and consequently the activity of the other cells (Wang et al., 2014; Shi et al., 2017). MSCs indeed can be induced by cytokines such as IFNγ, tumor necrosis factor alpha (TNFα), and interleukin (IL)-1 to release molecules that are involved in regulation of the innate and adaptive response of the immune system, such as prostaglandin E2 (PGE2) and indoleamine 2,3-dioxygenase (IDO), and chemokines, such as C-X-C chemokine motif ligand (CXCL)-9, CXCL10, and CXLC11, inducible nitric oxide synthase (iNOS) and other catabolites, such as adenosine (Ren et al., 2008).

Furthermore, TA-MSCs release mitogens such as epithelial growth factor (EGF), hepatocyte growth factor (HGF), EGF family members, insulin-like growth factor-1 (IGF-1), and different members of the fibroblast growth factor (FGF) family, which are able to directly stimulate cancer cell proliferation, and they release chemokines, such as stromal cell-derived factor-1 (SDF-1/CXCL12) able to trigger the recruitment of progenitor cells or the proliferation of stem cells (Franco et al., 2010; Rasanen and Vaheri, 2010). As a matter of fact, a role for the stromal compartment in chemo-resistance either through cell–cell contact or in a paracrine fashion has been proposed (Castells et al., 2012), and has also been shown to sustain cancer stemness (Su et al., 2018).

Moreover, TA-MSCs can promote EMT favoring tumor spread and the extracellular matrix remodeling (Ghosh et al., 2020). Increased invasiveness has been reported in 3D *in vitro* models based on heterotypic cell culture systems where TA-MSCs modulate ECM stiffness via matrix synthesis and remodeling, thus supporting tumor cell mobility and invasion (Akerfelt et al., 2015; Chung et al., 2017; Lee et al., 2018).

A pro-metastatic phenotype for TA-MSCs has been reported to be dependent on a variety factors, such as CXCL12, shown to favor EMT in prostate cancer (Jung et al., 2013) and, together with IGF-1, to select for clones with bone-metastatic ability in breast cancer (Zhang et al., 2013). In addition, a tumor–MSC–tumor feedback loop involving CCL5 signaling (Karnoub et al., 2007) and enhanced collagen deposition via discoidin domain receptor (DDR)-2 on TA-MSCs (Gonzalez et al., 2017) has been shown to stimulate breast cancer motility, invasion, and fibronectin alignment (Erdogan et al., 2017); enhance TA-MSC engulfment by breast cancer cells linked to enhanced metastatic potential (Chen et al., 2019); and enhance TA-MSC-derived exosomes by cancer stem cell thus boosting glioma aggressiveness (Figueroa et al., 2017). Exosomes, the smallest (30–150 nm) member of the extracellular vesicle family, represent a carrier for miRNA and other paracrine signals or factors capable of modulating the response of cancer cells and the immune system in the TME (Figueroa et al., 2017; Biswas et al., 2019).

The following sections of this review will focus on the ability of MSCs to affect tumorigenesis through their interplay and modulation of immune cells within the TME.

#### MSCs and Cells of the Innate Immune System

MSCs in the TME play a relevant role in favoring the recruitment and differentiation of different subsets of innate immune cells. *In vitro* and *in vivo* studies have shown that MSCs isolated from different sources are able to affect monocyte differentiation toward antigen-presenting cells, skewing them from the canonical inflammatory phenotype to acquire features typical of tolerogenic cells (Spaggiari et al., 2009; Magatti et al., 2015; Chiossone et al., 2016). Furthermore, MSCs skew the differentiation of monocyte-derived dendritic cells toward MDSCs through the action of the secreted growth-regulated oncogene (GRO-y) chemokine (Chen et al., 2013). Moreover, MSCs can also trigger the expansion of MDSCs through the release of high amounts of HGFs, demonstrating that the mechanism of function of the MSCs was not strictly associated to the release of immunomodulatory cytokines or chemokines, but was also related to the release of mitogens.

Others have highlighted the relevant role that cancer cells have in educating the stromal component associated to the tumor (TA-MSCs or CAFs), consequently influencing their properties. For example, lymphoma-associated MSCs can trigger the recruitment of neutrophils, monocytes, and macrophages to the TME through the release of high amounts of chemokine (C-C motif) ligand-2 (CCL2), CCL7, and CCL12, all of which are ligands of the CCR2 receptor that mediates chemotaxis and migration processes. The same effect was not observed when the experiments were performed using “non-tumor educated” bone marrow MSCs (Ren et al., 2012). The increased expression of the CCR2 ligand on BM-MSC was reported to be strictly related to the exposure to the inflammatory cytokine TNFα (Ren et al., 2012). In line with these observations, “tumor-educated” MSCs, and more specifically MSCs isolated from squamous cell lung carcinoma, became more strongly immunosuppressive in comparison to MSCs isolated from healthy tissues. Indeed, TA-MSCs were able to not only affect the phenotype but also decrease the cytotoxic activity of NK cells dampening their immune function (Galland et al., 2017). The immunosuppressive mechanisms, as illustrated by the type and quantity of immunosuppressive cytokines produced and the level of NK cell receptor ligands expressed, may differ between healthy and TA-MSCs, possibly as a function of the type of stimulatory microenvironment to which the cells are exposed (Galland et al., 2017). In addition, human and mouse TA-MSC exosomes were shown to accelerate breast cancer progression by inducing differentiation of MDSC into highly immunosuppressive M2-polarized macrophages (Biswas et al., 2019).

In quiescent tissues and in the absence of inflammatory stimuli, MSCs decrease or even temporarily lose their immunosuppressive features. Instead, in a TME that mimics tissue repair and contains a vast array of inflammatory cytokines derived from tumor cells, MSCs can be stimulated to release immunosuppressive molecules.

#### MSCs and Cells of the Adaptive Immune System

Adaptive immunity plays a fundamental role in controlling tumor progression. After tumor-associated macrophages, T and B lymphocytes are the second most frequent type of immune cell found within the tumor (Speiser et al., 2016; Donadon et al., 2017).

In fact, during tumor progression, the loss of immunogenicity by the tumor cells, together with the induction of peripheral tolerance, causes the tumor to evade the action of the cytotoxic component of the immune system (Palucka and Coussens, 2016).

The mechanism of induction of a peripheral tolerance is considered to be part of the normal control mechanisms of the inflammatory response; MSCs and the tumor stroma play a pivotal role in this regard. For example, bone marrow stromal cells have been shown to skew T lymphocyte toward Treg (Patel et al., 2014). Treg induction has been shown to occur through the release of TGFβ1, ultimately protecting breast cancer cells from an immune attack. Indeed, *in vitro* experiments confirmed the capacity of MSCs to affect the TME, creating an immune suppressive microenvironment, whereby BM-MSCs were able to protect T47D breast cancer cells from the cytotoxic activity of CD8 and NK cells by inducing Treg polarization (Patel et al., 2010).

In addition, BM-MSCs have been shown to reduce the expression of the chemokine CXCL12 by reducing the recruitment of peripheral blood mononuclear cells (PBMCs) to the tumor and by promoting the polarization of lymphocytes toward a Th2 subset with expansion of Treg cells (Patel et al., 2010). The suppressive effect of MSCs was demonstrated also in a murine melanoma model where subcutaneous injection of B16 melanoma cells with MSCs led to tumor growth in allogeneic mice. This effect was related to the capacity of MSCs to inhibit melanoma rejection, putatively through the induction of regulatory CD8^+^ T cells (Djouad et al., 2003).

On the other hand, the mesenchymal component was shown to inhibit the cytotoxic activity of CD8 lymphocytes in co-culture with BM-MSC by downregulation of HLA-I expressed by CaSki cervical carcinoma cells and downregulation of IL-10 expression (Montesinos et al., 2013). Interestingly, the elimination of CAFs using a FAP-targeting DNA vaccine in the 4T1 murine model of metastatic breast cancer switched the immune microenvironment of the tumor from a Th2 (anti-inflammatory) to a Th1 (pro-inflammatory) phenotype, indicating a key role for CAFs in polarizing the immune response to a pro-tumor type (Liao et al., 2009). Similar results were also obtained in a mouse model of breast cancer in which transplanted human MSCs significantly reduced CD3^+^ NKp46 NKT-like cells, increased CD4^+^ Foxp3 T cells and IL-10 expressing CD4^+^ cells, increased serum Th2 cytokines, and decreased serum Th1 cytokines (Ljujic et al., 2013). These results demonstrate that MSCs promote an immunosuppressive environment that ultimately favors breast tumor growth and metastasis in mice.

These studies provide evidence that MSC- and CAF-mediated suppression of adaptive immune cells enhance tumor growth.

#### MSC Crosstalk With Immune Cells Depends on Tumor Inflammatory Environment

Despite what has been highlighted so far about the role played by MSCs in the TME, MSCs can also exert an immunostimulatory effect triggering the immune response when the inflammatory conditions are suboptimal. An *in vitro* study demonstrated the capacity of antigen pulsed MSCs primed with low levels of IFN-γ to trigger the cytotoxic activity of CD8 T lymphocytes (Chan et al., 2006). Similarly, mouse BM-MSCs with reduced or genetically ablated iNOS production, treated with low concentrations of IFNγ and TNF, were found to enhance the proliferation of activated T cells (Li et al., 2012). Similar results were obtained also by another study showing that the development of B16 murine melanoma was accelerated when the melanoma cells were co-injected with MSCs pre-incubated with IFN-γ and TNF-α compared with controls. This effect was reverted when B16 cells were co-injected with iNOS-silenced MSCs (Han et al., 2011).

Importantly, iNOS^–/–^ MSCs significantly inhibited melanoma growth only in wild type, and not in immunodeficient mice, strongly suggesting the involvement of immune cells in the MSC-mediated anti-tumor effect (Li et al., 2012). The observed effects were related to the reduction of IDO and iNOS released by the primed MSCs (Li et al., 2012).

In addition, MSCs can respond to stimuli from the microenvironment by polarizing and acquiring peculiar properties. Initially, two different MSC phenotypes were identified: the proinflammatory MSC1 phenotype originated in response to the toll like receptor 4 (TLR4) priming and the anti-inflammatory MSC2 phenotype derived from the TLR3 priming (Patel et al., 2010). Nowadays, this vision is constantly evolving considering MSC plasticity and their ability to serve as “rheostat” cells capable of responding to the environmental stimulus by modifying the factors released. Indeed, low-dose and short-term TLR4 priming of MSCs provides the pro-inflammatory signature, characterized by a higher IL-6 and IL-8 secretion compared to the unstimulated MSC (Romieu-Mourez et al., 2009), and the TLR4-primed MSCs were unable to inhibit the proliferation of PBMCs (Waterman et al., 2010). This therapeutic approach, although fascinating and promising, requires further investigation.

## Perinatal MSC as Therapeutic Strategy in Cancer

In this section, we focus our attention on perinatal MSCs as exogenous MSCs that could be applied in anti-cancer therapy. Perinatal MSCs can be isolated from different placental regions, such as amniotic membrane (hAMSC), umbilical cord/Wharton’s jelly (UC-MSC/WJ-MSC), chorionic villi (CV-MSC), and maternal decidua (DMSC) (Silini et al., 2017a).

The benefits of using perinatal tissue include both technical and biological features, making this material a more valuable choice when compared to others. Technically, perinatal tissues present advantages in terms of tissue accessibility and MSC abundance with respect to adipose tissue and bone marrow. Normally considered medical waste, perinatal tissues (e.g., placenta) can be collected after normal delivery by non-invasive procedures.

Among the unique biological features that make perinatal MSCs valuable as anti-tumor therapeutic approach is their tumor tropism. For example, human amniotic fluid MSCs were shown able to migrate *in vivo* toward ovarian cancer (Chinnadurai et al., 2015) and bladder cancer, rat UC-MSCs were shown to home to mammary adenocarcinoma (Ganta et al., 2009) and Lewis lung carcinoma (Maurya et al., 2010), and human UC-MSCs were detected within or in close proximity to breast tumors in mice (Ayuzawa et al., 2009).

In addition, perinatal MSCs possess widely recognized intrinsic immune-regulatory properties and indeed they suppress T and B lymphocyte proliferation; inhibit the inflammatory cytokine production and functions of antigen-presenting cells (monocytes, dendritic cells, and macrophages), neutrophils, and NK cells; and enhance the formation of cells with regulatory activity such as Treg and M2 macrophages. Mainly due to these immunomodulatory properties, perinatal MSCs have been successfully exploited in the pre-clinical treatment of inflammatory and immune-based disorders and have been investigated in several clinical trials, such as in peripheral arterial disease and Crohn’s disease^1^.

On the other hand, similarly to BM-MSCs for which some immunogenic and immune-stimulatory activity has also been described (Magatti et al., 2018), low concentrations of both fetal (hAMSC, UC-MSC) and maternal (decidual) placental MSCs have been shown to stimulate the proliferation of PBMCs (Wolbank et al., 2007; Karlsson et al., 2012; Li et al., 2012; Papait et al., 2020). Therefore, in diseases such as cancer, where the stimulation of immune system has been proposed as an efficient therapeutic strategy (Ichim, 2005), understanding the mechanisms that underline the immune stimulation of perinatal MSCs is fundamental in order to exploit these properties for an anti-cancer therapy. Another important factor that requires investigation is the safety concern regarding the potential tumorigenicity of MSCs. Several studies have shown that placental MSCs do not form tumors when injected either subcutaneously or intravenously in mice (Rachakatla et al., 2007; Vulcano et al., 2016). In addition, MSCs from the amniotic membrane (Bonomi et al., 2015) and other tissues (Pessina et al., 2011; Bonomi et al., 2013) do not proliferate when loaded with the chemotherapeutic drug paclitaxel, which is relevant to suggest their lack of tumorigenesis and potentially safe application. Moreover, the identity of the bioactive factors that contribute to the anti- and pro-tumor actions (those produced by the MSC themselves or induced as bystander effects in the TME) remains another important aspect to be determined before clinical use of perinatal MSCs can be foreseen in the oncology field.

**FIGURE 2:**
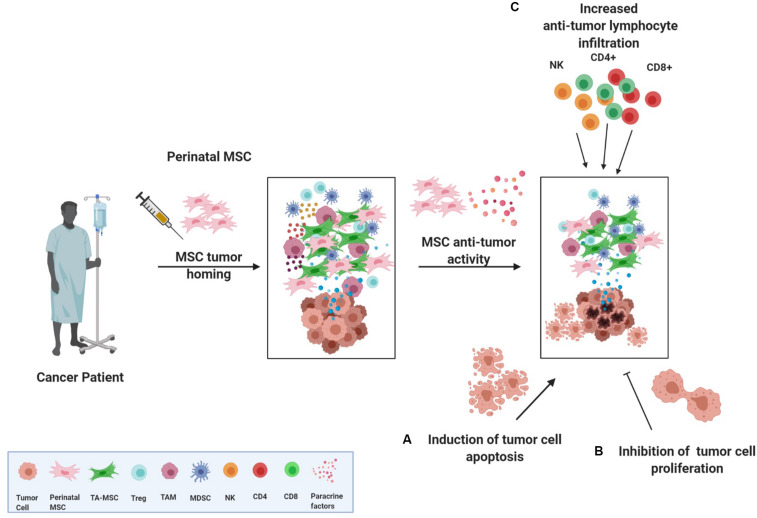
Perinatal MSCs and their anti-tumor actions. Despite controversial results obtained in investigating the action and the role of MSC in the TME, perinatal MSCs have been reported to exert antitumor actions. More specifically, once perinatal MSCs are injected in a cancer patient, they could home to the tumor and inflammatory site and re-educate the tumor microenvironment through three different actions: **(A)** induction of apoptosis of cancer cells, **(B)** inhibition of cancer cell proliferation, and **(C)** fostering the recruitment of CD4, CD8, and NK cells in the tumor mass, thus activating an anti-tumor response. TA-MSC, tumor associated mesenchymal stromal cell; Treg, T regulatory cell; TAM, tumor associated macrophage; MDSC, myeloid-derived suppressor cell; NK, natural killer cell.

The rationale for the application of perinatal MSCs as anti-tumor therapy relies on features such as (a) their ability to home to tumor sites, (b) their anti-proliferative action on various tumor cell lines *in vitro*, and (c) their ability to shape the inflammatory niche through their intrinsic immune regulatory or stimulatory abilities (Figure 2).

Perinatal MSCs have been reported to perform both tumor-supportive and tumor-restraining actions; however, whether this contrasting outcome delineates the versatile immune regulatory ability of MSCs has not been clarified yet.

To date, several *in vitro* and *in vivo* studies have highlighted the anti-tumor potential of perinatal MSCs and of their derivatives, such as conditioned medium (CM) generated from *in vitro* cell culture and extracellular vesicles (EVs) obtained from CM (Silini et al., 2017b).

UC-MSC/WJ-MSCs represent the most exploited perinatal MSCs in cancer. Many studies evaluated the effects of UC-MSCs, and their secreted factors, on breast cancer cells such as MDA-MB-231 and MCF-7, but with contrasting results. For example, UC-MSCs (Ayuzawa et al., 2009; Ganta et al., 2009; Chao et al., 2012; Ma et al., 2012) and their CM (Ayuzawa et al., 2009; Ganta et al., 2009; Gauthaman et al., 2012) have been shown to inhibit the *in vitro* proliferation of breast cancer cells such as MDA-MB-231 and MCF-7, while others reported that CM (Li et al., 2015) and EV from UC-MSCs (Zhou et al., 2019) promote the proliferation and migration of the same cells.

The anti-tumor actions of UC-MSC/WJ-MSCs have also been explored on cancer cell lines other than breast carcinoma. They have indeed been shown to exert *in vitro* anti-proliferative action on ovarian carcinomas (Gauthaman et al., 2012; Khalil et al., 2019) and osteosarcoma (MG-63) (Gauthaman et al., 2012). CM from UC-MSC/WJ-MSCs was found to be effective in inhibiting the growth of human glioma (U251) (Yang et al., 2014) and leukemia (K562) (Hendijani et al., 2015), and micro-vesicles from UC-MSC/WJ-MSC were reported to attenuate the proliferation of bladder tumor cells (T24) (Wu et al., 2013). Controversial actions have been reported for lung carcinoma; indeed, rat UC-MSCs reduced the growth of Lewis lung carcinoma (Maurya et al., 2010); however, CM from human WJ-MSCs did not affect the proliferation of both A549 lung cancer cells (Hendijani et al., 2015) and SPC-A-1 lung adenocarcinoma cells (Meng et al., 2019). In addition, EVs from human UC-MSCs increased proliferation and decreased apoptosis in lung adenocarcinoma cells (Dong et al., 2018) and those from human WJ-MSCs fostered the aggressiveness human renal cell carcinoma (786-0) cells (Du et al., 2014).

Pre-clinical tumor models have also been employed to assess the effect of perinatal MSCs on tumor growth and progression. Consistently with *in vitro* results, UC-MSC/WJ-MSCs and their derivatives were able to reduce tumor volume (Ganta et al., 2009; Maurya et al., 2010; Chao et al., 2012; Ma et al., 2012; Wu et al., 2013) and counteract lung metastatic growth of breast cancer lines (Ayuzawa et al., 2009; Ganta et al., 2009; Chao et al., 2012). Interestingly, Wu et al. (2013) observed that hWJ-MSC-derived micro-vesicles mixed with T24 tumor cells were more effective than hWJ-MSCs *per se* at reducing tumor volume and tumor incidence in a xenograft model of bladder cancer.

In agreement with the pro-tumor activity observed *in vitro*, UC-MSC/WJ-MSCs and derivatives did not reduce tumor volume (Meng et al., 2019) and even promoted the growth of lung adenocarcinoma (Dong et al., 2018) and of renal cell carcinoma (Du et al., 2014).

Controversial findings were also reported when perinatal cells different from UC-MSC/WJ-MSCs were used as anti-tumor therapy. For example, our group has demonstrated that MSCs derived from amniotic membrane (hAMSCs) inhibited the proliferation *in vitro* of various tumor cell lines, such as hematopoietic [lymphoid (KG1a, Jurkat), myeloid (KG1, U937)] and non-hematopoietic Girardi heart, Hela, and Saos tumor cells, both in contact and when cultured in a transwell system, indicating the involvement of secreted factors (Magatti et al., 2012). In line with our *in vitro* observations, others have shown that intra-tumor injection of hAMSCs inhibited growth and induced apoptosis of C6 glioma (Jiao et al., 2012). To the contrary, CM from hAMSCs was shown to promote the proliferation and migration of breast cell lines (Kim et al., 2015), lung adenocarcinoma cells (SPC-A-1), and gastric carcinoma cells (BGC-823) (Meng et al., 2019). Interestingly, the authors of this last study only partially confirmed their findings *in vivo* as hAMSCs were able to stimulate tumor growth when subcutaneously co-injected with SPC-A-1 or BGC-823 tumor cells, but not when injected intravenously 12 days after tumor cell injection when the tumor is well-established (Meng et al., 2019). Others instead observed no effects on tumor volume or mice survival when human amniotic fluid MSCs were iv-injected in bladder tumor-bearing animals (Bitsika et al., 2012).

Both CV-MSCs and DMSCs have been shown to possess anti-tumor properties. Indeed, CV-MSCs were shown to inhibit the proliferation and migration of a triple-negative breast cancer cell line (MDA-MB231) *in vitro* (Alshareeda et al., 2018), and DMSCs reduced the growth of rat primary mammary tumors induced by N-nitroso-N-methylurea and the development of secondary tumors (Vegh et al., 2013).

The snapshot of pre-clinical trials outlined here underlines the high degree of plasticity that endows MSCs from perinatal tissues. Indeed, in some of the cases reported above, despite the application of similar experimental conditions (e.g., the same type of perinatal MSC, the same type of target tumor, etc.), MSCs display both anti-tumor and pro-tumor activities, but at present, with current knowledge, it is difficult to say which activity prevails. Nevertheless, there are many technical and biological aspects to be considered when comparing independent studies, such as differences in isolation/culture protocols, presence of xenogenic, possibly immunogenic, factors (such as BSA), in culture media and in the experimental methods of both primary cells and commercial cell lines, as well as variation in oncogenic and mutational pattern, and in the expression of specific receptors by cancer cell lines. In addition, different ratios of MSCs–tumor cells (Silini et al., 2017a) and timing of MSC delivery into tumors (Klopp et al., 2011) constitute an important argument in the comparison of different studies. Hence, all these details play a major role in defining MSC properties and impact the final outcome.

Given the pleiotropic nature of perinatal MSCs, different mechanisms have been suggested as mediators of the cells’ effect on tumor development. The main mechanism proposed is represented by direct impairment of tumor growth. This process involves tumor cell cycle arrest, accompanied by a decrease in the expression of cyclin A and cyclin-dependent kinase-2 (CDK2) (Maurya et al., 2010; Ma et al., 2012; Magatti et al., 2012; Wu et al., 2013), and/or promotion of tumor cell apoptosis, with upregulation of apoptotic genes (caspase-3 and caspase-9), and downregulation of anti-apoptotic genes [survivin and X-linked inhibitor of apoptosis protein (XIAP)] (Wu et al., 2013). Perinatal MSCs can attenuate/inhibit the PI3K/AKT pathway, an important regulator of cellular metabolism and the survival and proliferation of tumor cells (Ayuzawa et al., 2009; Ganta et al., 2009; Ma et al., 2012; Wu et al., 2013). An interesting study by Chao et al. (2012) reported that hUC-MSCs exert their anti-tumor action either by secreting bioactive factors or by becoming internalized by tumor cells to form a cell-in-cell structure resulting in tumor cell apoptosis. To our knowledge, very few papers investigated whether perinatal MSCs can affect the inflammatory environment generated by tumor cells.

This is in part due to the use of tumor-induced animal models where human tumor cells (or tissues) are transplanted in immune deficient mice [athymic (nude) mice or severe combined immunodeficiency (SCID)]. These mice lack a normal immune response in order to allow for tumor growth and avoid tumor rejection. However, these conditions do not allow for a complete understanding of the interactions between tumor and immune cells, and the contribution of immune cells in response to therapy with perinatal MSC.

To this regard, one study analyzed the impact of perinatal MSCs on cytokines, growth factors, and chemokines secreted by tumor cells. In this study, supernatants collected from the co-culture of MSCs (hUC-MSCs or hAMSCs) and SPC-A1 lung adenocarcinoma cells revealed that inflammatory factors [granulocyte stimulating factor (G-CSF), granulocyte macrophage colony stimulating factor (GM-CSF), intracellular adhesion molecule (ICAM)-1, IL-1a, IL-1b, IL-1ra, IL-8, tissue inhibitor of metalloproteinases (TIMP)-2, IL-10, IL-16, and IL-6sR], growth factors [bone morphogenetic protein (BMP)4, platelet-derived growth factor (PDGF)-AA, PDGFBB, vascular endothelial growth factor (VEGF), EGFR, insulin like growth factor binding protein (IGFBP)2, IGFBP3 and bFGF], and chemokines [CXCL6, CXCL16, IL-9, IL-18 BPa, leukemia inhibitory factor (LIF), Lymphotactin, macrophage-derived chemokine (MDC), macrophage migration inhibitory factor (MIF), macrophage inflammatory protein (MIP)-3a, growth related oncogene (GRO) and SDF-1a] were remarkably altered after co-culturing, suggesting that these factors were involved in the regulation of MSC activities on SPC-A-1 cells. Among the downregulated cytokines, the reduction of VEGF, PDGF, and IL-6sR was correlated with anti-migration and anti-angiogenic actions exerted by perinatal MSCs on tumor cells (Meng et al., 2019). Another study demonstrated that intra-tumor injection of rat UC-MSCs significantly attenuated tumor growth and concomitantly increased intra-tumor infiltration of CD4^+^ and CD8^+^ T cells and NK cells and decreased levels of intra-tumor macrophages and Treg cells (Kawabata et al., 2013). These findings suggested that rat UC-MSCs can exert anti-tumor action by enhancing host tumor immune responses via promoting MCP-1-mediated recruitment of cytotoxic immune cells in tumor tissues.

## Conclusion

The TME has a decisive role in tumor progression and more specifically immune cells within the TME have the power to either promote or inhibit tumor growth.

In regenerative medicine, MSCs have long been investigated as a therapeutic strategy, and their contribution to improved outcome has largely been attributed to their unique ability to modulate immune cells. Thus, it comes natural to believe that MSCs, whether local, recruited, or exogenously administered, could re-educate immune cells within the TME. The question is what determines their ability to either favor immunosurveillance and thus inhibit tumor growth, or favor immune editing and thus support tumor growth. This dual role remains an intense area of investigation and is confounded by the plasticity of MSCs and their ability to be molded by the TME milieu.

When considering exogenous MSCs as an anti-cancer strategy, perinatal MSCs have unique features. Perinatal MSCs are largely obtained from the placenta, and the placenta’s contribution to the development and growth of a semi-allogeneic fetus during pregnancy favors the idea that cells from the placenta possess intrinsic, peculiar immunological characteristics. Furthermore, the lack of ethical concerns, the easy isolation and handling, and the low/absent expression of human leukocyte antigens and co-stimulatory molecules make perinatal MSCs interesting candidates, especially for allogeneic transplantation. However, their contribution in cancer field and specifically their ability to interact and modulate the immune system of the TME need more investigation since they are no exception to dual roles that MSCs have in tumor progression. Finally, there is also a need to develop immune-competent models in order to better understand the interactions between MSCs and immune cells in the TME.

## Author Contributions

FS and AP: writing – original draft preparation. AC, MM, AS, and OP: writing – review and editing. OP: supervision and final approval of the manuscript. All authors contributed to the article and approved the submitted version.

## Conflict of Interest

The authors declare that the research was conducted in the absence of any commercial or financial relationships that could be construed as a potential conflict of interest.

## Footnotes

1 https://clinicaltrials.gov/

## References

[B1] Akerfelt M. Bayramoglu N. Robinson S. Toriseva M. Schukov H. P. Harma V. (2015). Automated tracking of tumor-stroma morphology in microtissues identifies functional targets within the tumor microenvironment for therapeutic intervention. *Oncotarget* 6 30035–30056. 10.18632/oncotarget.5046 26375443 PMC4745780

[B2] Allen E. Jabouille A. Rivera L. B. Lodewijckx I. Missiaen R. Steri V. (2017). Combined antiangiogenic and anti-PD-L1 therapy stimulates tumor immunity through HEV formation. *Sci. Transl. Med.* 9:eaak9679. 10.1126/scitranslmed.aak9679 28404866 PMC5554432

[B3] Alshareeda A. T. Rakha E. Alghwainem A. Alrfaei B. (2018). The effect of human placental chorionic villi derived mesenchymal stem cell on triple-negative breast cancer hallmarks. *PLoS One* 13:e0207593. 10.1371/journal.pone.0207593 30458011 PMC6245746

[B4] Arina A. Idel C. Hyjek E. M. Alegre M. L. Wang Y. Bindokas V. P. (2016). Tumor-associated fibroblasts predominantly come from local and not circulating precursors. *Proc. Natl. Acad. Sci. U.S.A.* 113 7551–7556. 10.1073/pnas.1600363113 27317748 PMC4941507

[B5] Augsten M. (2014). Cancer-associated fibroblasts as another polarized cell type of the tumor microenvironment. *Front. Oncol.* 4:62. 10.3389/fonc.2014.00062 24734219 PMC3973916

[B6] Avila-Ibarra L. R. Mora-Garcia M. L. Garcia-Rocha R. Hernandez-Montes J. Weiss-Steider B. Montesinos J. J. (2019). Mesenchymal stromal cells derived from normal cervix and cervical cancer tumors increase CD73 expression in cervical cancer cells through TGF-beta1 production. *Stem Cells Dev.* 28 477–488. 10.1089/scd.2018.0183 30696359

[B7] Ayuzawa R. Doi C. Rachakatla R. S. Pyle M. M. Maurya D. K. Troyer D. (2009). Naive human umbilical cord matrix derived stem cells significantly attenuate growth of human breast cancer cells *in vitro* and *in vivo*. *Cancer Lett.* 280 31–37. 10.1016/j.canlet.2009.02.011 19285791 PMC2914472

[B8] Barcellos-de-Souza P. Comito G. Pons-Segura C. Taddei M. L. Gori V. Becherucci V. (2016). Mesenchymal stem cells are recruited and activated into carcinoma-associated fibroblasts by prostate cancer microenvironment-derived TGF-beta1. *Stem Cells* 34 2536–2547. 10.1002/stem.2412 27300750

[B9] Barnes T. A. Amir E. (2018). HYPE or HOPE: the prognostic value of infiltrating immune cells in cancer. *Br. J. Cancer* 118:e5. 10.1038/bjc.2017.417 29315291 PMC5785752

[B10] Behnan J. Isakson P. Joel M. Cilio C. Langmoen I. A. Vik-Mo E. O. (2014). Recruited brain tumor-derived mesenchymal stem cells contribute to brain tumor progression. *Stem Cells* 32 1110–1123. 10.1002/stem.1614 24302539

[B11] Bhagat T. D. Von Ahrens D. Dawlaty M. Zou Y. Baddour J. Achreja A. (2019). Lactate-mediated epigenetic reprogramming regulates formation of human pancreatic cancer-associated fibroblasts. *eLife* 8:e50663. 10.7554/eLife.50663 PMC6874475 31663852

[B12] Biswas S. Mandal G. Roy Chowdhury S. Purohit S. Payne K. K. Anadon C. (2019). Exosomes produced by mesenchymal stem cells drive differentiation of myeloid cells into immunosuppressive M2-polarized macrophages in breast cancer. *J. Immunol*. 203 3447–3460. 10.4049/jimmunol.1900692 31704881 PMC6994919

[B13] Bitsika V. Roubelakis M. G. Zagoura D. Trohatou O. Makridakis M. Pappa K. I. (2012). Human amniotic fluid-derived mesenchymal stem cells as therapeutic vehicles: a novel approach for the treatment of bladder cancer. *Stem Cells Dev.* 21 1097–1111. 10.1089/scd.2011.0151 21988169

[B14] Bochet L. Lehuede C. Dauvillier S. Wang Y. Y. Dirat B. Laurent V. (2013). Adipocyte-derived fibroblasts promote tumor progression and contribute to the desmoplastic reaction in breast cancer. *Cancer Res.* 73 5657–5668. 10.1158/0008-5472.can-13-0530 23903958

[B15] Bonomi A. Cocce V. Cavicchini L. Sisto F. Dossena M. Balzarini P. (2013). Adipose tissue-derived stromal cells primed *in vitro* with paclitaxel acquire anti-tumor activity. *Int. J. Immunopathol. Pharmacol.* 26(1 Suppl.), 33–41. 10.1177/03946320130260s105 24046947

[B16] Bonomi A. Silini A. Vertua E. Signoroni P. B. Cocce V. Cavicchini L. (2015). Human amniotic mesenchymal stromal cells (hAMSCs) as potential vehicles for drug delivery in cancer therapy: an *in vitro* study. *Stem Cell Res. Ther.* 6:155. 10.1186/s13287-015-0140-z PMC4552458 26315881

[B17] Borriello L. Nakata R. Sheard M. A. Fernandez G. E. Sposto R. Malvar J. (2017). Cancer-associated fibroblasts share characteristics and protumorigenic activity with mesenchymal stromal cells. *Cancer Res.* 77 5142–5157. 10.1158/0008-5472.can-16-2586 28687621 PMC5600847

[B18] Cano E. Gebala V. Gerhardt H. (2017). Pericytes or mesenchymal stem cells: Is that the question? *Cell Stem Cell* 20 296–297. 10.1016/j.stem.2017.02.005 28257708

[B19] Caplan A. I. Dennis J. E. (2006). Mesenchymal stem cells as trophic mediators. *J. Cell. Biochem.* 98 1076–1084. 10.1002/jcb.20886 16619257

[B20] Castells M. Thibault B. Delord J. P. Couderc B. (2012). Implication of tumor microenvironment in chemoresistance: tumor-associated stromal cells protect tumor cells from cell death. *Int. J. Mol. Sci.* 13 9545–9571. 10.3390/ijms13089545 22949815 PMC3431813

[B21] Chan J. L. Tang K. C. Patel A. P. Bonilla L. M. Pierobon N. Ponzio N. M. (2006). Antigen-presenting property of mesenchymal stem cells occurs during a narrow window at low levels of interferon-gamma. *Blood* 107 4817–4824. 10.1182/blood-2006-01-0057 16493000 PMC1895812

[B22] Chao K. C. Yang H. T. Chen M. W. (2012). Human umbilical cord mesenchymal stem cells suppress breast cancer tumourigenesis through direct cell-cell contact and internalization. *J. Cell. Mol. Med.* 16 1803–1815. 10.1111/j.1582-4934.2011.01459.x 21973190 PMC3822693

[B23] Chen H. W. Chen H. Y. Wang L. T. Wang F. H. Fang L. W. Lai H. Y. (2013). Mesenchymal stem cells tune the development of monocyte-derived dendritic cells toward a myeloid-derived suppressive phenotype through growth-regulated oncogene chemokines. *J. Immunol.* 190 5065–5077. 10.4049/jimmunol.1202775 23589610

[B24] Chen Y. C. Gonzalez M. E. Burman B. Zhao X. Anwar T. Tran M. (2019). Mesenchymal stem/stromal cell engulfment reveals metastatic advantage in breast cancer. *Cell Rep.* 27 3916–3926.e5. 10.1016/j.celrep.2019.05.084 31242423 PMC6657699

[B25] Cheng J. Ye H. Liu Z. Xu C. Zhang Z. Liu Y. (2013). Platelet-derived growth factor-BB accelerates prostate cancer growth by promoting the proliferation of mesenchymal stem cells. *J. Cell. Biochem.* 114 1510–1518. 10.1002/jcb.24492 23297038

[B26] Chinnadurai R. Copland I. B. Ng S. Garcia M. Prasad M. Arafat D. (2015). Mesenchymal stromal cells derived from Crohn’s patients deploy indoleamine 2,3-dioxygenase-mediated immune suppression, independent of autophagy. *Mol. Ther.* 23 1248–1261. 10.1038/mt.2015.67 25899824 PMC4817795

[B27] Chiossone L. Conte R. Spaggiari G. M. Serra M. Romei C. Bellora F. (2016). Mesenchymal stromal cells induce peculiar alternatively activated macrophages capable of dampening both innate and adaptive immune responses. *Stem Cells)* 34 1909–1921. 10.1002/stem.2369 27015881

[B28] Chung B. Esmaeili A. A. Gopalakrishna-Pillai S. Murad J. P. Andersen E. S. Kumar Reddy N. (2017). Human brain metastatic stroma attracts breast cancer cells via chemokines CXCL16 and CXCL12. *NPJ Breast Cancer* 3:6. 10.1038/s41523-017-0008-8 PMC5460196 28649646

[B29] Coffman L. G. Pearson A. T. Frisbie L. G. Freeman Z. Christie E. Bowtell D. D. (2019). Ovarian carcinoma-associated mesenchymal stem cells arise from tissue-specific normal stroma. *Stem Cells* 37 257–269. 10.1002/stem.2932 30353617 PMC6392140

[B30] Coussens L. M. Werb Z. (2002). Inflammation and Cancer. *Nature* 420 860–867. 12490959 10.1038/nature01322 PMC2803035

[B31] Crisan M. Yap S. Casteilla L. Chen C. W. Corselli M. Park T. S. (2008). A perivascular origin for mesenchymal stem cells in multiple human organs. *Cell Stem Cell* 3 301–313. 10.1016/j.stem.2008.07.003 18786417

[B32] da Silva Meirelles L. Chagastelles P. C. Nardi N. B. (2006). Mesenchymal stem cells reside in virtually all post-natal organs and tissues. *J. Cell Sci.* 119(Pt 11), 2204–2213. 10.1242/jcs.02932 16684817

[B33] de Visser K. E. Eichten A. Coussens L. M. (2006). Paradoxical roles of the immune system during cancer development. *Nat. Rev. Cancer* 6 24–37. 10.1038/nrc1782 16397525

[B34] Dirat B. Bochet L. Dabek M. Daviaud D. Dauvillier S. Majed B. (2011). Cancer-associated adipocytes exhibit an activated phenotype and contribute to breast cancer invasion. *Cancer Res.* 71 2455–2465. 10.1158/0008-5472.can-10-3323 21459803

[B35] Direkze N. C. Forbes S. J. Brittan M. Hunt T. Jeffery R. Preston S. L. (2003). Multiple organ engraftment by bone-marrow-derived myofibroblasts and fibroblasts in bone-marrow-transplanted mice. *Stem Cells* 21 514–520. 10.1634/stemcells.21-5-514 12968105

[B36] Direkze N. C. Hodivala-Dilke K. Jeffery R. Hunt T. Poulsom R. Oukrif D. (2004). Bone marrow contribution to tumor-associated myofibroblasts and fibroblasts. *Cancer Res.* 64 8492–8495. 10.1158/0008-5472.can-04-1708 15574751

[B37] Djouad F. Plence P. Bony C. Tropel P. Apparailly F. Sany J. (2003). Immunosuppressive effect of mesenchymal stem cells favors tumor growth in allogeneic animals. *Blood* 102 3837–3844. 10.1182/blood-2003-04-1193 12881305

[B38] Dominici M. Le Blanc K. Mueller I. Slaper-Cortenbach I. Marini F. Krause D. (2006). Minimal criteria for defining multipotent mesenchymal stromal cells. The International Society for Cellular Therapy position statement. *Cytotherapy* 8 315–317. 10.1080/14653240600855905 16923606

[B39] Donadon M. Hudspeth K. Cimino M. Di Tommaso L. Preti M. Tentorio P. (2017). Increased infiltration of natural killer and T cells in colorectal liver metastases improves patient overall survival. *J. Gastrointest. Surg.* 21 1226–1236. 10.1007/s11605-017-3446-6 28536806

[B40] Dong L. Pu Y. Zhang L. Qi Q. Xu L. Li W. (2018). Human umbilical cord mesenchymal stem cell-derived extracellular vesicles promote lung adenocarcinoma growth by transferring miR-410. *Cell Death Dis.* 9:218. 10.1038/s41419-018-0323-5 PMC5833395 29440630

[B41] Du T. Ju G. Wu S. Cheng Z. Cheng J. Zou X. (2014). Microvesicles derived from human Wharton’s jelly mesenchymal stem cells promote human renal cancer cell growth and aggressiveness through induction of hepatocyte growth factor. *PLoS One* 9:e96836. 10.1371/journal.pone.0096836 24797571 PMC4010513

[B42] Dumont N. Liu B. Defilippis R. A. Chang H. Rabban J. T. Karnezis A. N. (2013). Breast fibroblasts modulate early dissemination, tumorigenesis, and metastasis through alteration of extracellular matrix characteristics. *Neoplasia* 15 249–262. 23479504 10.1593/neo.121950 PMC3593149

[B43] Dunn G. P. Old L. J. Schreiber R. D. (2004). The immunobiology of cancer immunosurveillance and immunoediting. *Immunity* 21 137–148. 10.1016/j.immuni.2004.07.017 15308095

[B44] Erdogan B. Ao M. White L. M. Means A. L. Brewer B. M. (2017). Cancer-associated fibroblasts promote directional cancer cell migration by aligning fibronectin. *J. Cell Biol.* 216 3799–3816. 10.1083/jcb.201704053 29021221 PMC5674895

[B45] Erez N. Truitt M. Olson P. Arron S. T. Hanahan D. (2010). Cancer-associated fibroblasts are activated in incipient neoplasia to orchestrate tumor-promoting inflammation in an NF-kappaB-dependent manner. *Cancer Cell* 17 135–147. 10.1016/j.ccr.2009.12.041 20138012

[B46] Fiaschi T. Marini A. Giannoni E. Taddei M. L. Gandellini P. De Donatis A. (2012). Reciprocal metabolic reprogramming through lactate shuttle coordinately influences tumor-stroma interplay. *Cancer Res.* 72 5130–5140. 10.1158/0008-5472.can-12-1949 22850421

[B47] Figueroa J. Phillips L. M. Shahar T. Hossain A. Gumin J. Kim H. (2017). Exosomes from glioma-associated mesenchymal stem cells increase the tumorigenicity of glioma stem-like cells via transfer of miR-1587. *Cancer Res.* 77 5808–5819. 10.1158/0008-5472.can-16-2524 28855213 PMC5668150

[B48] Franco O. E. Shaw A. K. Strand D. W. Hayward S. W. (2010). Cancer associated fibroblasts in cancer pathogenesis. *Semin. Cell Dev. Biol.* 21 33–39. 10.1016/j.semcdb.2009.10.010 19896548 PMC2823834

[B49] Friedenstein A. J. Deriglasova U. F. Kulagina N. N. Panasuk A. F. Rudakowa S. F. Luria E. A. (1974). Precursors for fibroblasts in different populations of hematopoietic cells as detected by the *in vitro* colony assay method. *Exp. Hematol.* 2 83–92. 4455512

[B50] Friedenstein A. J. Petrakova K. V. Kurolesova A. I. Frolova G. P. (1968). Heterotopic of bone marrow. Analysis of precursor cells for osteogenic and hematopoietic tissues. *Transplantation* 6 230–247. 5654088

[B51] Galland S. Vuille J. Martin P. Letovanec I. Caignard A. Fregni G. (2017). Tumor-derived mesenchymal stem cells use distinct mechanisms to block the activity of natural killer cell subsets. *Cell Rep.* 20 2891–2905. 10.1016/j.celrep.2017.08.089 28930684

[B52] Ganta C. Chiyo D. Ayuzawa R. Rachakatla R. Pyle M. Andrews G. (2009). Rat umbilical cord stem cells completely abolish rat mammary carcinomas with no evidence of metastasis or recurrence 100 days post-tumor cell inoculation. *Cancer Res.* 69 1815–1820. 10.1158/0008-5472.can-08-2750 19244122

[B53] Gauthaman K. Yee F. C. Cheyyatraivendran S. Biswas A. Choolani M. Bongso A. (2012). Human umbilical cord Wharton’s jelly stem cell (hWJSC) extracts inhibit cancer cell growth *in vitro*. *J. Cell. Biochem.* 113 2027–2039. 10.1002/jcb.24073 22275115

[B54] Ghosh D. Mejia Pena C. Quach N. Xuan B. Lee A. H. Dawson M. R. (2020). Senescent mesenchymal stem cells remodel extracellular matrix driving breast cancer cells to a more-invasive phenotype. *J. Cell Sci.* 133:jcs232470. 10.1242/jcs.232470 31932504 PMC6983709

[B55] Girardi M. Glusac E. Filler R. B. Roberts S. J. Propperova I. Lewis J. (2003). The distinct contributions of murine T cell receptor (TCR)gammadelta+ and TCRalphabeta+ T cells to different stages of chemically induced skin cancer. *J. Exp. Med.* 198 747–755. 10.1084/jem.20021282 12953094 PMC2194182

[B56] Gonzalez M. E. Martin E. E. Anwar T. Arellano-Garcia C. Medhora N. Lama A. (2017). Mesenchymal stem cell-induced DDR2 mediates stromal-breast cancer interactions and metastasis growth. *Cell Rep.* 18 1215–1228. 10.1016/j.celrep.2016.12.079 28147276 PMC5332146

[B57] Greten F. R. Grivennikov S. I. (2019). Inflammation and cancer: triggers, mechanisms, and consequences. *Immunity* 51 27–41. 10.1016/j.immuni.2019.06.025 31315034 PMC6831096

[B58] Han Z. Tian Z. Lv G. Zhang L. Jiang G. Sun K. (2011). Immunosuppressive effect of bone marrow-derived mesenchymal stem cells in inflammatory microenvironment favours the growth of B16 melanoma cells. *J. Cell. Mol. Med.* 15 2343–2352. 10.1111/j.1582-4934.2010.01215.x 21091630 PMC3822946

[B59] Hendijani F. Javanmard S. H. Sadeghi-aliabadi H. (2015). Human Wharton’s jelly mesenchymal stem cell secretome display antiproliferative effect on leukemia cell line and produce additive cytotoxic effect in combination with doxorubicin. *Tissue Cell* 47 229–234. 10.1016/j.tice.2015.01.005 25779671

[B60] Hossain A. Gumin J. Gao F. Figueroa J. Shinojima N. Takezaki T. (2015). Mesenchymal stem cells isolated from human gliomas increase proliferation and maintain stemness of glioma stem cells through the IL-6/gp130/STAT3 pathway. *Stem Cells* 33 2400–2415. 10.1002/stem.2053 25966666 PMC4509942

[B61] Hughes R. M. Simons B. W. Khan H. Miller R. Kugler V. Torquato S. (2019). Asporin restricts mesenchymal stromal cell differentiation, alters the tumor microenvironment, and drives metastatic progression. *Cancer Res.* 79 3636–3650. 10.1158/0008-5472.can-18-2931 31123087 PMC6734938

[B62] Ichim C. V. (2005). Revisiting immunosurveillance and immunostimulation: implications for cancer immunotherapy. *J. Transl. Med.* 3:8. 10.1186/1479-5876-3-8 PMC549049 15698481

[B63] Jiao H. Guan F. Yang B. Li J. Song L. Hu X. (2012). Human amniotic membrane derived-mesenchymal stem cells induce C6 glioma apoptosis *in vivo* through the Bcl-2/caspase pathways. *Mol. Biol. Rep.* 39 467–473. 10.1007/s11033-011-0760-z 21556762

[B64] Jung Y. Kim J. K. Shiozawa Y. Wang J. Mishra A. Joseph J. (2013). Recruitment of mesenchymal stem cells into prostate tumours promotes metastasis. *Nat. Commun.* 4:1795. 10.1038/ncomms2766 PMC3649763 23653207

[B65] Kalluri R. (2016). The biology and function of fibroblasts in cancer. *Nat. Rev. Cancer* 16 582–598. 10.1038/nrc.2016.73 27550820

[B66] Kansy B. A. Dissmann P. A. Hemeda H. Bruderek K. Westerkamp A. M. Jagalski V. (2014). The bidirectional tumor–mesenchymal stromal cell interaction promotes the progression of head and neck cancer. *Stem Cell Res. Ther.* 5:95. 10.1186/scrt484 25115189 PMC4535379

[B67] Karlsson H. Erkers T. Nava S. Ruhm S. Westgren M. Ringden O. (2012). Stromal cells from term fetal membrane are highly suppressive in allogeneic settings *in vitro*. *Clin. Exp. Immunol.* 167 543–555. 10.1111/j.1365-2249.2011.04540.x 22288598 PMC3374287

[B68] Karnoub A. E. Dash A. B. Vo A. P. Sullivan A. Brooks M. W. Bell G. W. (2007). Mesenchymal stem cells within tumour stroma promote breast cancer metastasis. *Nature* 449 557–563. 10.1038/nature06188 17914389

[B69] Kawabata A. Ohta N. Seiler G. Pyle M. M. Ishiguro S. Zhang Y. Q. (2013). Naive rat umbilical cord matrix stem cells significantly attenuate mammary tumor growth through modulation of endogenous immune responses. *Cytotherapy* 15 586–597. 10.1016/j.jcyt.2013.01.006 23474329 PMC3652627

[B70] Khalil C. Moussa M. Azar A. Tawk J. Habbouche J. Salameh R. (2019). Anti-proliferative effects of mesenchymal stem cells (MSCs) derived from multiple sources on ovarian cancer cell lines: an *in-vitro* experimental study. *J. Ovarian Res.* 12:70. 10.1186/s13048-019-0546-9 PMC6660927 31351482

[B71] Kidd S. Spaeth E. Watson K. Burks J. Lu H. Klopp A. (2012). Origins of the tumor microenvironment: quantitative assessment of adipose-derived and bone marrow-derived stroma. *PLoS One* 7:e30563. 10.1371/journal.pone.0030563 22363446 PMC3282707

[B72] Kim S. H. Bang S. H. Kang S. Y. Park K. D. Eom J. H. Oh I. U. (2015). Human amniotic membrane-derived stromal cells (hAMSC) interact depending on breast cancer cell type through secreted molecules. *Tissue Cell* 47 10–16. 10.1016/j.tice.2014.10.003 25441616

[B73] Klopp A. H. Gupta A. Spaeth E. Andreeff M. Marini F. III (2011). Concise review: Dissecting a discrepancy in the literature: do mesenchymal stem cells support or suppress tumor growth? *Stem Cells* 29 11–19. 10.1002/stem.559 21280155 PMC3059412

[B74] Kojima Y. Acar A. Eaton E. N. Mellody K. T. Scheel C. Ben-Porath I. (2010). Autocrine TGF-beta and stromal cell-derived factor-1 (SDF-1) signaling drives the evolution of tumor-promoting mammary stromal myofibroblasts. *Proc. Natl. Acad. Sci. U.S.A.* 107 20009–20014. 10.1073/pnas.1013805107 21041659 PMC2993333

[B75] Landskron G. De la Fuente M. Thuwajit P. Thuwajit C. Hermoso M. A. (2014). Chronic inflammation and cytokines in the tumor microenvironment. *J. Immunol. Res.* 2014:149185. 10.1155/2014/149185 PMC4036716 24901008

[B76] Lee J. Shin D. Roh J.-L. (2018). Development of an *in vitro* cell-sheet cancer model for chemotherapeutic screening. *Theranostics* 8 3964–3973. 10.7150/thno.26439 30083273 PMC6071526

[B77] Li T. Zhang C. Ding Y. Zhai W. Liu K. Bu F. (2015). Umbilical cord-derived mesenchymal stem cells promote proliferation and migration in MCF-7 and MDA-MB-231 breast cancer cells through activation of the ERK pathway. *Oncol. Rep.* 34 1469–1477. 10.3892/or.2015.4109 26151310

[B78] Li W. Ren G. Huang Y. Su J. Han Y. Li J. (2012). Mesenchymal stem cells: a double-edged sword in regulating immune responses. *Cell Death Differ.* 19 1505–1513. 10.1038/cdd.2012.26 22421969 PMC3422473

[B79] Liao D. Luo Y. Markowitz D. Xiang R. Reisfeld R. A. (2009). Cancer associated fibroblasts promote tumor growth and metastasis by modulating the tumor immune microenvironment in a 4T1 murine breast cancer model. *PLoS One* 4:e7965. 10.1371/journal.pone.0007965 19956757 PMC2775953

[B80] Ljujic B. Milovanovic M. Volarevic V. Murray B. Bugarski D. Przyborski S. (2013). Human mesenchymal stem cells creating an immunosuppressive environment and promote breast cancer in mice. *Sci. Rep.* 3:2298. 10.1038/srep02298 PMC3725512 23892388

[B81] Lohr M. Schmidt C. Ringel J. Kluth M. Muller P. Nizze H. (2001). Transforming growth factor-beta1 induces desmoplasia in an experimental model of human pancreatic carcinoma. *Cancer Res.* 61 550–555. 11212248

[B82] Lynch M. D. Watt F. M. (2018). Fibroblast heterogeneity: implications for human disease. *J. Clin. Invest.* 128 26–35. 10.1172/jci93555 29293096 PMC5749540

[B83] Lyssiotis C. A. Kimmelman A. C. (2017). Metabolic interactions in the tumor microenvironment. *Trends Cell Biol.* 27 863–875. 10.1016/j.tcb.2017.06.003 28734735 PMC5814137

[B84] Ma Y. Hao X. Zhang S. Zhang J. (2012). The *in vitro* and *in vivo* effects of human umbilical cord mesenchymal stem cells on the growth of breast cancer cells. *Breast Cancer Res. Treat.* 133 473–485. 10.1007/s10549-011-1774-x 21947651

[B85] Magatti M. Caruso M. De Munari S. Vertua E. De D. Manuelpillai U. (2015). Human amniotic membrane-derived mesenchymal and epithelial cells exert different effects on monocyte-derived dendritic cell differentiation and function. *Cell Transplant.* 24 1733–1752. 10.3727/096368914x684033 25259480

[B86] Magatti M. De Munari S. Vertua E. Parolini O. (2012). Amniotic membrane-derived cells inhibit proliferation of cancer cell lines by inducing cell cycle arrest. *J. Cell. Mol. Med.* 16 2208–2218. 10.1111/j.1582-4934.2012.01531.x 22260183 PMC3822990

[B87] Magatti M. Vertua E. Cargnoni A. Silini A. Parolini O. (2018). The immunomodulatory properties of amniotic cells: the two sides of the coin. *Cell Transplant.* 27 31–44. 10.1177/0963689717742819 29562786 PMC6434482

[B88] Mantovani A. Allavena P. Sica A. Balkwill F. (2008). Cancer-related inflammation. *Nature* 454 436–444. 18650914 10.1038/nature07205

[B89] Marrazzo P. Paduano F. Palmieri F. Marrelli M. Tatullo M. (2016). Highly efficient *in vitro* reparative behaviour of dental pulp stem cells cultured with standardised platelet lysate supplementation. *Stem Cells Int.* 2016:7230987. 10.1155/2016/7230987 PMC5059612 27774106

[B90] Martin-Orozco N. Muranski P. Chung Y. Yang X. O. Yamazaki T. Lu S. (2009). T helper 17 cells promote cytotoxic T cell activation in tumor immunity. *Immunity* 31 787–798. 10.1016/j.immuni.2009.09.014 19879162 PMC2787786

[B91] Maurya D. K. Doi C. Kawabata A. Pyle M. M. King C. Wu Z. (2010). Therapy with un-engineered naive rat umbilical cord matrix stem cells markedly inhibits growth of murine lung adenocarcinoma. *BMC Cancer* 10:590. 10.1186/1471-2407-10-590 21029413 PMC2988749

[B92] McLean K. Gong Y. Choi Y. Deng N. Yang K. Bai S. (2011). Human ovarian carcinoma-associated mesenchymal stem cells regulate cancer stem cells and tumorigenesis via altered BMP production. *J. Clin. Invest.* 121 3206–3219. 10.1172/jci45273 21737876 PMC3148732

[B93] Meng M. Y. Li L. Wang W. J. Liu F. F. Song J. Yang S. L. (2019). Assessment of tumor promoting effects of amniotic and umbilical cord mesenchymal stem cells *in vitro* and *in vivo*. *J. Cancer Res. Clin. Oncol.* 145 1133–1146. 10.1007/s00432-019-02859-6 30805774 PMC6482126

[B94] Mishra P. J. Mishra P. J. Humeniuk R. Medina D. J. Alexe G. Mesirov J. P. (2008). Carcinoma-associated fibroblast-like differentiation of human mesenchymal stem cells. *Cancer Res.* 68 4331–4339. 10.1158/0008-5472.can-08-0943 18519693 PMC2725025

[B95] Montesinos J. J. Mora-Garcia Mde L. Mayani H. Flores-Figueroa E. Garcia-Rocha R. Fajardo-Orduna G. R. (2013). *In vitro* evidence of the presence of mesenchymal stromal cells in cervical cancer and their role in protecting cancer cells from cytotoxic T cell activity. *Stem Cells Dev.* 22 2508–2519. 10.1089/scd.2013.0084 23656504 PMC3761677

[B96] Motz G. T. Coukos G. (2013). Deciphering and reversing tumor immune suppression. *Immunity* 39 61–73. 10.1016/j.immuni.2013.07.005 23890064 PMC3782392

[B97] Naour A. L. Prat M. Thibault B. Mevel R. Lemaitre L. Leray H. (2019). Tumor cells educate mesenchymal stromal cells to release chemoprotective and immunomodulatory factors. *J. Mol. Cell Biol.* 12 202–215. 10.1093/jmcb/mjz090 31504643 PMC7181721

[B98] O’Donnell J. S. Teng M. W. L. Smyth M. J. (2019). Cancer immunoediting and resistance to T cell-based immunotherapy. *Nat. Rev. Clin. Oncol.* 16 151–167. 10.1038/s41571-018-0142-8 30523282

[B99] Oloyo A. K. Ambele M. A. Pepper M. S. (2018). Contrasting views on the role of mesenchymal stromal/stem cells in tumour growth: a systematic review of experimental design. *Adv. Exp. Med. Biol.* 1083 103–124. 10.1007/5584_2017_118 29139088

[B100] Palucka A. K. Coussens L. M. (2016). The basis of oncoimmunology. *Cell* 164 1233–1247. 10.1016/j.cell.2016.01.049 26967289 PMC4788788

[B101] Papait A. Vertua E. Magatti M. (2020). Mesenchymal stromal cells from fetal and maternal placenta possess key similarities and differences: potential implications for their applications in regenerative medicine. *Cells* 9:127. 10.3390/cells9010127 31935836 PMC7017205

[B102] Patel S. A. Dave M. A. Bliss S. A. Giec-Ujda A. B. Bryan M. Pliner L. F. (2014). Treg/Th17 polarization by distinct subsets of breast cancer cells is dictated by the interaction with mesenchymal stem cells. *J. Cancer Stem Cell Res.* 2014:e1003. 10.14343/JCSCR.2014.2e1003 PMC4334154 25705705

[B103] Patel S. A. Meyer J. R. Greco S. J. Corcoran K. E. Bryan M. Rameshwar P. (2010). Mesenchymal stem cells protect breast cancer cells through regulatory T cells: role of mesenchymal stem cell-derived TGF-beta. *J. Immunol*. 184 5885–5894. 10.4049/jimmunol.0903143 20382885

[B104] Pelizzo G. Veschi V. Mantelli M. Croce S. Di Benedetto V. D’Angelo P. (2018). Microenvironment in neuroblastoma: isolation and characterization of tumor-derived mesenchymal stromal cells. *BMC Cancer* 18:1176. 10.1186/s12885-018-5082-2 30482160 PMC6260687

[B105] Pessina A. Bonomi A. Cocce V. Invernici G. Navone S. Cavicchini L. (2011). Mesenchymal stromal cells primed with paclitaxel provide a new approach for cancer therapy. *PLoS One* 6:e28321. 10.1371/journal.pone.0028321 22205945 PMC3243689

[B106] Poggi A. Varesano S. Zocchi M. R. (2018). How to hit mesenchymal stromal cells and make the tumor microenvironment immunostimulant rather than immunosuppressive. *Front. Immunol.* 9:262. 10.3389/fimmu.2018.00262 29515580 PMC5825917

[B107] Quante M. Tu S. P. Tomita H. Gonda T. Wang S. S. Takashi S. (2011). Bone marrow-derived myofibroblasts contribute to the mesenchymal stem cell niche and promote tumor growth. *Cancer Cell* 19 257–272. 10.1016/j.ccr.2011.01.020 21316604 PMC3060401

[B108] Rachakatla R. S. Marini F. Weiss M. L. Tamura M. Troyer D. (2007). Development of human umbilical cord matrix stem cell-based gene therapy for experimental lung tumors. *Cancer Gene Ther.* 14 828–835. 10.1038/sj.cgt.7701077 17599089

[B109] Radisky D. C. Kenny P. A. Bissell M. J. (2007). Fibrosis and cancer: do myofibroblasts come also from epithelial cells via EMT? *J. Cell. Biochem.* 101 830–839. 10.1002/jcb.21186 17211838 PMC2838476

[B110] Rasanen K. Vaheri A. (2010). Activation of fibroblasts in cancer stroma. *Exp. Cell Res.* 316 2713–2722. 10.1016/j.yexcr.2010.04.032 20451516

[B111] Raz Y. Cohen N. Shani O. Bell R. E. Novitskiy S. V. Abramovitz L. (2018). Bone marrow-derived fibroblasts are a functionally distinct stromal cell population in breast cancer. *J. Exp. Med.* 215 3075–3093. 10.1084/jem.20180818 30470719 PMC6279405

[B112] Ren G. Zhang L. Zhao X. Xu G. Zhang Y. Roberts A. I. (2008). Mesenchymal stem cell-mediated immunosuppression occurs via concerted action of chemokines and nitric oxide. *Cell Stem Cell* 2 141–150. 10.1016/j.stem.2007.11.014 18371435

[B113] Ren G. Zhao X. Wang Y. Zhang X. Chen X. Xu C. (2012). CCR2-dependent recruitment of macrophages by tumor-educated mesenchymal stromal cells promotes tumor development and is mimicked by TNFalpha. *Cell Stem Cell* 11 812–824. 10.1016/j.stem.2012.08.013 23168163 PMC3518598

[B114] Romieu-Mourez R. Francois M. Boivin M. N. Bouchentouf M. Spaner D. E. Galipeau J. (2009). Cytokine modulation of TLR expression and activation in mesenchymal stromal cells leads to a proinflammatory phenotype. *J. Immunol.* 182 7963–7973. 10.4049/jimmunol.0803864 19494321

[B115] Sacchetti B. Funari A. Michienzi S. Di Cesare S. Piersanti S. Saggio I. (2007). Self-renewing osteoprogenitors in bone marrow sinusoids can organize a hematopoietic microenvironment. *Cell* 131 324–336. 10.1016/j.cell.2007.08.025 17956733

[B116] Sacchetti B. Funari A. Remoli C. Giannicola G. Kogler G. Liedtke S. (2016). No identical “Mesenchymal Stem Cells” at different times and sites: human committed progenitors of distinct origin and differentiation potential are incorporated as adventitial cells in microvessels. *Stem Cell Rep.* 6 897–913. 10.1016/j.stemcr.2016.05.011 27304917 PMC4912436

[B117] Sautes-Fridman C. Petitprez F. Calderaro J. Fridman W. H. (2019). Tertiary lymphoid structures in the era of cancer immunotherapy. *Nat. Rev. Cancer* 19 307–325. 10.1038/s41568-019-0144-6 31092904

[B118] Shahar T. Rozovski U. Hess K. R. Hossain A. Gumin J. Gao F. (2017). Percentage of mesenchymal stem cells in high-grade glioma tumor samples correlates with patient survival. *Neuro Oncol.* 19 660–668. 28453745 10.1093/neuonc/now239 PMC5464439

[B119] Sharon Y. Raz Y. Cohen N. Ben-Shmuel A. Schwartz H. Geiger T. (2015). Tumor-derived osteopontin reprograms normal mammary fibroblasts to promote inflammation and tumor growth in breast cancer. *Cancer Res.* 75 963–973. 10.1158/0008-5472.can-14-1990 25600648

[B120] Shi Y. Du L. Lin L. Wang Y. (2017). Tumour-associated mesenchymal stem/stromal cells: emerging therapeutic targets. *Nat. Rev. Drug Discov.* 16 35–52. 10.1038/nrd.2016.193 27811929

[B121] Silini A. R. Cancelli S. Signoroni P. B. Cargnoni A. Magatti M. Parolini O. (2017a). The dichotomy of placenta-derived cells in cancer growth. *Placenta* 59 154–162. 10.1016/j.placenta.2017.05.011 28545651

[B122] Silini A. R. Magatti M. Cargnoni A. Parolini O. (2017b). Is immune modulation the mechanism underlying the beneficial effects of amniotic cells and their derivatives in regenerative medicine? *Cell Transplant.* 26 531–539. 10.3727/096368916x693699 27938500 PMC5661217

[B123] Smyth M. J. Thia K. Y. Street S. E. Cretney E. Trapani J. A. Taniguchi M. (2000). Differential tumor surveillance by natural killer (NK) and NKT cells. *J. Exp. Med.* 191 661–668. 10.1084/jem.191.4.661 10684858 PMC2195840

[B124] Soundararajan M. Kannan S. (2018). Fibroblasts and mesenchymal stem cells: Two sides of the same coin? *J. Cell. Physiol.* 233 9099–9109. 10.1002/jcp.26860 29943820

[B125] Spaeth E. L. Dembinski J. L. Sasser A. K. Watson K. Klopp A. Hall B. (2009). Mesenchymal stem cell transition to tumor-associated fibroblasts contributes to fibrovascular network expansion and tumor progression. *PLoS One* 4:e4992. 10.1371/journal.pone.0004992 19352430 PMC2661372

[B126] Spaeth E. L. Labaff A. M. Toole B. P. Klopp A. Andreeff M. Marini F. C. (2013). Mesenchymal CD44 expression contributes to the acquisition of an activated fibroblast phenotype via TWIST activation in the tumor microenvironment. *Cancer Res.* 73 5347–5359. 10.1158/0008-5472.can-13-0087 23838935 PMC3767181

[B127] Spaggiari G. M. Abdelrazik H. Becchetti F. Moretta L. (2009). MSCs inhibit monocyte-derived DC maturation and function by selectively interfering with the generation of immature DCs: central role of MSC-derived prostaglandin E2. *Blood* 113 6576–6583. 10.1182/blood-2009-02-203943 19398717

[B128] Speiser D. E. Ho P. C. Verdeil G. (2016). Regulatory circuits of T cell function in cancer. *Nat. Rev. Immunol.* 16 599–611. 10.1038/nri.2016.80 27526640

[B129] Su S. Chen J. Yao H. Liu J. Yu S. Lao L. (2018). CD10(+)GPR77(+) cancer-associated fibroblasts promote cancer formation and chemoresistance by sustaining cancer stemness. *Cell* 172 841–856.e16. 10.1016/j.cell.2018.01.009 29395328

[B130] Svensson A. Ramos-Moreno T. Eberstal S. Scheding S. Bengzon J. (2017). Identification of two distinct mesenchymal stromal cell populations in human malignant glioma. *J. Neurooncol.* 131 245–254. 10.1007/s11060-016-2302-y 27757723 PMC5306185

[B131] Toullec A. Gerald D. Despouy G. Bourachot B. Cardon M. Lefort S. (2010). Oxidative stress promotes myofibroblast differentiation and tumour spreading. *EMBO Mol. Med.* 2 211–230. 10.1002/emmm.201000073 20535745 PMC3377319

[B132] Turley S. J. Cremasco V. Astarita J. L. (2015). Immunological hallmarks of stromal cells in the tumour microenvironment. *Nat. Rev. Immunol.* 15 669–682. 10.1038/nri3902 26471778

[B133] Udagawa T. Puder M. Wood M. Schaefer B. C. D’Amato R. J. (2006). Analysis of tumor-associated stromal cells using SCID GFP transgenic mice: contribution of local and bone marrow-derived host cells. *FASEB J.* 20 95–102. 10.1096/fj.04-3669com 16394272

[B134] Vegh I. Grau M. Gracia M. Grande J. de la Torre P. Flores A. I. (2013). Decidua mesenchymal stem cells migrated toward mammary tumors *in vitro* and *in vivo* affecting tumor growth and tumor development. *Cancer Gene Ther.* 20 8–16. 10.1038/cgt.2012.71 23037810

[B135] Vignaud J. M. Marie B. Klein N. Plenat F. Pech M. Borrelly J. (1994). The role of platelet-derived growth factor production by tumor-associated macrophages in tumor stroma formation in lung cancer. *Cancer Res.* 54 5455–5463. 7923179

[B136] Vulcano F. Milazzo L. Ciccarelli C. Eramo A. Sette G. Mauro A. (2016). Wharton’s jelly mesenchymal stromal cells have contrasting effects on proliferation and phenotype of cancer stem cells from different subtypes of lung cancer. *Exp. Cell Res.* 345 190–198. 10.1016/j.yexcr.2016.06.003 27343631

[B137] Wang Y. Chen X. Cao W. Shi Y. (2014). Plasticity of mesenchymal stem cells in immunomodulation: pathological and therapeutic implications. *Nat. Immunol.* 15 1009–1016. 10.1038/ni.3002 25329189

[B138] Waterman R. S. Tomchuck S. L. Henkle S. L. Betancourt A. M. (2010). A new mesenchymal stem cell (MSC) paradigm: polarization into a pro-inflammatory MSC1 or an Immunosuppressive MSC2 phenotype. *PLoS One* 5:e10088. 10.1371/journal.pone.0010088 20436665 PMC2859930

[B139] Wolbank S. Peterbauer A. Fahrner M. Hennerbichler S. van Griensven M. Stadler G. (2007). Dose-dependent immunomodulatory effect of human stem cells from amniotic membrane: a comparison with human mesenchymal stem cells from adipose tissue. *Tissue Eng.* 13 1173–1183. 10.1089/ten.2006.0313 17518752

[B140] Wu S. Ju G. Q. Du T. Zhu Y. J. Liu G. H. (2013). Microvesicles derived from human umbilical cord Wharton’s jelly mesenchymal stem cells attenuate bladder tumor cell growth *in vitro* and *in vivo*. *PLoS One* 8:e61366. 10.1371/journal.pone.0061366 23593475 PMC3625149

[B141] Yang C. Lei D. Ouyang W. Ren J. Li H. Hu J. (2014). Conditioned media from human adipose tissue-derived mesenchymal stem cells and umbilical cord-derived mesenchymal stem cells efficiently induced the apoptosis and differentiation in human glioma cell lines *in vitro*. *Biomed. Res. Int.* 2014:109389. 10.1155/2014/109389 PMC4058294 24971310

[B142] Yuen G. J. Demissie E. Pillai S. (2016). B lymphocytes and cancer: a love-hate relationship. *Trends Cancer* 2 747–757. 10.1016/j.trecan.2016.10.010 28626801 PMC5472356

[B143] Zeisberg E. M. Potenta S. Xie L. Zeisberg M. Kalluri R. (2007). Discovery of endothelial to mesenchymal transition as a source for carcinoma-associated fibroblasts. *Cancer Res.* 67 10123–10128. 10.1158/0008-5472.can-07-3127 17974953

[B144] Zhang X. Hu F. Li G. Li G. Yang X. Liu L. (2018). Human colorectal cancer-derived mesenchymal stem cells promote colorectal cancer progression through IL-6/JAK2/STAT3 signaling. *Cell Death Dis.* 9:25. 10.1038/s41419-017-0176-3 PMC5833830 29348540

[B145] Zhang X. H. Jin X. Malladi S. Zou Y. Wen Y. H. Brogi E. (2013). Selection of bone metastasis seeds by mesenchymal signals in the primary tumor stroma. *Cell* 154 1060–1073. 10.1016/j.cell.2013.07.036 23993096 PMC3974915

[B146] Zhang Y. Daquinag A. Traktuev D. O. Amaya-Manzanares F. Simmons P. J. March K. L. (2009). White adipose tissue cells are recruited by experimental tumors and promote cancer progression in mouse models. *Cancer Res.* 69 5259–5266. 10.1158/0008-5472.can-08-3444 19491274 PMC3857703

[B147] Zhang Y. Daquinag A. C. Amaya-Manzanares F. Sirin O. Tseng C. Kolonin M. G. (2012). Stromal progenitor cells from endogenous adipose tissue contribute to pericytes and adipocytes that populate the tumor microenvironment. *Cancer Res.* 72:5198. 10.1158/0008-5472.can-12-0294 23071132

[B148] Zhou X. Li T. Chen Y. Zhang N. Wang P. Liang Y. (2019). Mesenchymal stem cellderived extracellular vesicles promote the *in vitro* proliferation and migration of breast cancer cells through the activation of the ERK pathway. *Int. J. Oncol.* 54 1843–1852. 30864702 10.3892/ijo.2019.4747

